# Pollen Competition as a Reproductive Isolation Barrier Represses Transgene Flow between Compatible and Co-Flowering Citrus Genotypes

**DOI:** 10.1371/journal.pone.0025810

**Published:** 2011-10-03

**Authors:** Elsa Pons, Antonio Navarro, Patrick Ollitrault, Leandro Peña

**Affiliations:** 1 Centro de Protección Vegetal y Biotecnología, Instituto Valenciano de Investigaciones Agrarias (IVIA), Moncada, Valencia, Spain; 2 Centre de Coopération Internationale en Recherche Agronomique pour le Développement (CIRAD), UPR Amélioration Génétique des Espèces à Multiplication Végétative, Montpellier, France; University College London, United Kingdom

## Abstract

**Background/Objective:**

Despite potential benefits granted by genetically modified (GM) fruit trees, their release and commercialization raises concerns about their potential environmental impact, and the transfer via pollen of transgenes to cross-compatible cultivars is deemed to be the greatest source for environmental exposure. Information compiled from field trials on GM trees is essential to propose measures to minimize the transgene dispersal. We have conducted a field trial of seven consecutive years to investigate the maximum frequency of pollen-mediated crop-to-crop transgene flow in a citrus orchard, and its relation to the genetic, phenological and environmental factors involved.

**Methodology/Principal Findings:**

Three different citrus genotypes carrying the *uidA* (GUS) tracer marker gene (pollen donors) and a non-GM self-incompatible contiguous citrus genotype (recipient) were used in conditions allowing natural entomophilous pollination to occur. The examination of 603 to 2990 seeds per year showed unexpectedly low frequencies (0.17–2.86%) of transgene flow. Paternity analyses of the progeny of subsets of recipient plants using 10 microsatellite (SSR) loci demonstrated a higher mating competence of trees from another non-GM pollen source population that greatly limited the mating chance of the contiguous cross-compatible and flowering-synchronized transgenic pollen source. This mating superiority could be explained by a much higher pollen competition capacity of the non-GM genotypes, as was confirmed through mixed-hand pollinations.

**Conclusions/Significance:**

Pollen competition strongly contributed to transgene confinement. Based on this finding, suitable isolation measures are proposed for the first time to prevent transgene outflow between contiguous plantings of citrus types that may be extendible to other entomophilous transgenic fruit tree species.

## Introduction

The progressive increase in the global area and number of GM crops has lead to numerous empirical studies on transgene flow in field trials aimed at developing containment strategies, which are required by regulators and policy makers to legislate, on a case-by-case basis, how deliberate releases should be performed. Containment could be important to protect the rights of the owner of the transgenic variety and of GM-free growers and to avoid the unintended release of certain transgenic traits to other cultivars or to wild relatives [1], [2]. Most of these investigations have so far been carried out in annual crops [3], [4], while research in perennial species is still scarce or is focused on contemporary gene flow based on the genetic structure of natural populations [5]–[8]. Thus, it is necessary to carry out transgene flow studies specifically in trees because their long life and complex reproductive biology may have significant effects on the extent of transgene dispersal.

Citrus is the most extensively produced fruit-tree crop in the world [9]. Commercial citrus genotypes are subjected to important biotic stresses, which are only partially controlled by the application of pesticides and, in many instances, limit the use of certain rootstocks and/or varieties. At the same time, markets demand fresh fruit and juice of increasing quality. In this context, the main focus of citrus breeding programs has been disease resistance plus fruit quality. However, improvement of citrus by conventional breeding is constrained by genetic crossing barriers, such as self and cross incompatibility, high heterozygosity, long juvenile periods, and facultative apomixis and sterility [10]. Genetic engineering (GE) could circumvent some of these limitations, especially by bypassing the long crossing cycles of tree breeding programs, without the complications of linkage drag. Moreover, it allows improvement of citrus varieties that are not amenable to breeding, like sweet oranges and grapefruits. Furthermore, GE is the only technology that enables gene transfer between unrelated organisms, even if they belong to widely divergent taxa, offering promising prospects in disease resistance approaches, especially when resistance sources are not present in reproductively compatible relatives. Thus, though there are no commercial GM citrus crops yet, genetic transformation is considered an essential tool in many current improvement programs, and experimental field trials are underway in several countries [11].

Cross-pollination in citrus is accomplished by insects, and honeybees are the most successful pollinators [12]. In insect-pollinated plants, pollen dispersal is generally the main component of gene flow [13]. The potential for pollen-based gene flow depends on the geographic distribution of the different compatible species (wild or crop) present in the area of study. In all citrus-production areas of the world, except East Asia, it is unlikely that transgenic plants could become feral populations because there are virtually no wild sympatric citrus species and relatives. However, cross-pollination between conventional citrus cultivars and transgenic citrus genotypes would be theoretically possible in many cases if they are grown in the same production areas. The presence of transgenic seeds in non-transgenic fruits as a result of effective cross-pollination could be a matter of concern. Although seeds in citrus are never consumed deliberately, their adventitious presence in non-GM fruits could cause problems related to consumer acceptance, and it may have implications on the marketability of the fruit, especially if organic fruit-growing orchards are exposed [14]. For the specific case of self-incompatible, cross-compatible mandarins and mandarin hybrids, this problem is not contemplated because the presence of seeds in the fruit already represents a marketability problem, so different cultural strategies are commonly used to avoid cross-pollination with sympatric citrus cultivars. From an agronomic viewpoint, there is no concern over the adventitious propagation of GM citrus cultivars through escaped seeds because commercial citrus varieties are exclusively propagated by grafting adult vegetative buds onto juvenile rootstocks. In the incidental case that transgenic seedlings germinated in an orchard, they would be removed by farmers. Moreover, these seedlings would never flower before being removed because citrus seedlings need several years to start flowering [15]. Information about pollen-mediated crop-to-crop gene flow from a GM citrus cultivar is therefore required to estimate the likelihood of the adventitious presence of GM seeds in non-GM citrus varieties grown in the same area.

In entomophilous species, the physical distance between the pollen source and sink is one of the most important factors determining the distribution of frequency and maximum dispersal distances of gene flow [16]. In fact, it is well known that bees in fruit tree orchards restrict their activity to single or adjacent plants [17], resulting in increased pollination between neighboring trees, e.g., in lychees [18], avocado [19], apples [20], almonds [21], citrus [22] and other tree species [23]. In all of these species, the maximum frequency of gene flow was adjacent to the source and rapidly declined with distance, often describing a marked leptokurtic curve [24].

Based on this finding, we designed an experimental field trial that involved the release of GM citrus trees with the objective of measuring during seven consecutive years the frequency of pollen-mediated transgene flow (PMTF) from GM lines to contiguous recipient trees under open pollination (OP) conditions. Three different citrus genotypes (sweet orange, citrange and lime) carrying the β-glucuronidase gene (*uid*A), which served in this study as marker to track gene transfer, were used as pollen donors, and clementine, a self-incompatible mandarin type, was used as the recipient.

Although recent studies demonstrate that bees have the potential to move pollen over several kilometers, the probability of pollen movement is very low if patches are more than 50 m away [25], and these rare outcrossing events contribute little to adventitious GM presence in non-GM receptor crops. Therefore, field assessment of the ‘extreme cases’ in which GM and non-GM citrus are cultivated adjacently is an essential first step for a thorough evaluation of gene flow and its potential consequences. Additionally, the influence of the diverse floral neighborhood on transgene flow frequency between sexually compatible and flowering-synchronized species located in close proximity was also assessed. The role of the floral neighborhood as a possible isolation barrier between GM and non-GM crops is investigated here for the first time, providing valuable information for properly designing future field trials for efficient GM containment. The study site where the experimental field is located represents a collection of genetic resources of citrus, such as various widely diverse cultivars and breeding materials, which allows estimating the frequency and range of gene flow from different pollen sources by paternity analysis of progeny from OP recipients with the assistance of specific molecular markers.

The objectives of this study were (1) to estimate the frequency of PMTF from three different GM citrus types to a non-GM citrus variety planted adjacently as an edge; (2) to assess the role of the surrounding flora as isolation barrier between co-flowering and compatible transgenic pollen donors and recipients through estimation of the mating success and gene flow patterns from different pollen sources within the study site; (3) to elucidate isolation mechanisms to explain how pollen donors showing higher mating success can limit PMTF; and (4) to propose containment strategies to repress transgene pollen dispersal from citrus (and other fruit) orchards.

## Materials and Methods

### Plant materials

Eight independent transgenic lines of three citrus genotypes with a different genetic background were used as potential pollen donors in this work: Pineapple sweet orange (*Citrus sinensis* L. Osb.; named P1 to P8), Carrizo citrange (*C. sinensis* L. Osb. x *Poncirus trifoliata* L. Raf.; named C1 to C8) and Mexican lime (*C. aurantifolia* (Christm.) Swing.; named L1 to L8). All transgenic lines carried the *35S::uidA::Nos* (GUSINT) and *Nos*::*nptII::Nos* marker transgenes, providing constitutive GUS expression and resistance to kanamycin, respectively. The *uidA* transgene was used as a marker to track gene flow. The transgenic lines used were selected based on their high-level transgene expression and low copy number of transgene insertions (ranging from 1 to 4, depending on the line) [26]. Three control lines (one per genotype, named PC, CC and LC) were also used in the current study as non-transgenic pollen donors. Trees of the self-incompatible and monoembryonic citrus genotype Clemenules clementine served as pollen recipients for monitoring PMTF.

### Experimental field design

**Figure 1:**
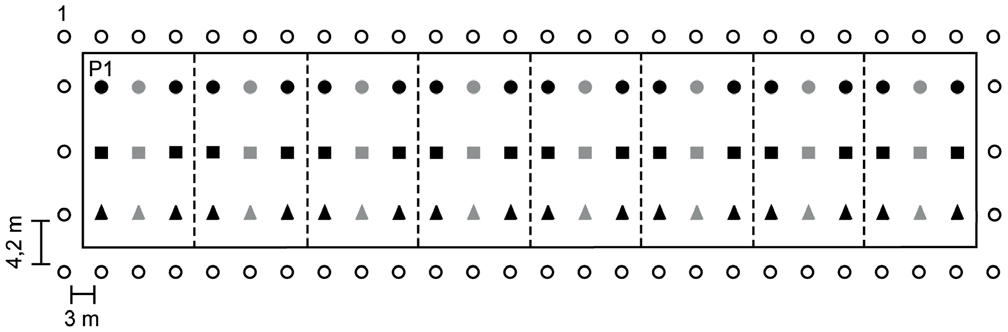
Schematic diagram of the experimental field trial. It consisted of 130 trees, planted in rows along the transgenic (T) plot, including 16 transgenic plants of Pineapple sweet orange (black circles), 16 transgenic plants of Carrizo citrange (black squares) and 16 transgenic plants of Mexican lime (black triangles) (2 plants each from 8 independent transgenic lines numbered from 1 to 8, from left to right). In addition, there were 8 non-transgenic control plants from each genotype individually interspersed between the two plants from each transgenic line and represented by grey figures. Fifty-eight non-transgenic Clemenules clementine trees planted along an external edge (white circles; numbered in increasing order going clockwise) were used as the pollen recipients to estimate transgene flow frequencies.

The gene flow experiment was conducted for seven production seasons (from 2001 to 2007) at an experimental field named T plot, located at the Instituto Valenciano de Investigaciones Agrarias, Spain (latitude 39°35”N, longitude 0°23”W and altitude 50 m; typical Mediterranean climate). The field study was designed to evaluate the short-distance PMTF from transgenic to non-transgenic citrus plants, that is, the maximum expected dispersal frequency. The T plot, with an extension of 1.638 m^2^, contained 130 adult trees distributed in rows, as described in Fig. 1. The pollen-donor genotypes (transgenic and control lines) were planted at the center, while 58 non-transgenic recipient clementine trees were planted on an external edge. All scion types were grafted onto Carrizo citrange rootstocks and grown in a loamy clay soil with drip irrigation. The field was managed as for normal citrus cultivation. No treatments were performed to control bees and pollinators in general. Visual surveys showed that the number of open flowers from pollen donors and recipient trees as well as the number of bees at the study site during the flowering periods greatly exceeded the amounts needed to ensure natural cross-pollination every year (Fig. S1).

### Determination of PMTF frequencies

Fruit samples of every open-pollinated (OP) recipient clementine tree were collected annually. At least 10 randomly selected fruits per tree were harvested when the fruits were fully mature. Seeds were extracted from fruits, counted and tested for GUS expression. A histochemical GUS assay was performed on seeds that were cut to provide substrate penetration. A sample of seeds from a transgenic citrus line was used as the positive control (Fig. S2A). The PMTF frequency was calculated annually as the percentage of GUS-positive (transgenic) seeds over the total number of seeds analyzed, and we assumed that this frequency was the maximum achievable for our experimental conditions due to the proximity of the recipient trees to the transgenic pollen source.

To validate the method used to determine the PMTF frequency, seedlings from seeds of an array of randomly selected OP recipient trees were tested for transgene expression and integration over 2 years (2005–2006). Seeds were sown on seedbeds containing steam-sterilized artificial soil mix suitable for growing citrus and under regular greenhouse conditions. The greenhouse-grown seedlings were assessed through histochemical GUS assays of the leaves (Fig. S2B,C) and PCR analysis for the *uidA* transgene. For PCR analysis, DNA was extracted from 20 mg of leaves according to [27]. Standard PCR techniques were used to detect the *uid*A transgene. The primers used to amplify the transgenic DNA fragment were GUS-up (5’-ggtgggaaagcgcgttacaag-3’) and GUS-down (5′-tggattccggcatagttaaa-3’). The reactions were performed in 30 cycles of 0.50 min at 95°C, 0.50 min at 58°C and 1 min at 72°C. The PCR products were detected by electrophoresis using 1% agarose-ethidium bromide gels. The DNA was stored at −20°C for further microsatellite (SSR; Simple Sequence Repeat) analyses.

### Flowering synchrony, pollen viability and cross-compatibility studies

To check the flowering synchrony between the pollen donor and recipient genotypes, the phenology of all trees in the T Plot was studied in 2005 and 2006 from the start of flowering to the initiation of fruit set. Phenological calendars were established for each genotype by weekly observation and recording of the predominant phenological stages of trees, following the BBCH codifications [28]. Mexican limes were excluded from this study because they tend to show sparse flowering over the year, which implies that throughout the year, almost all phenological stages can be found in a tree at the same time.

Pollen viability of all pollen donors of the T Plot (transgenic and control lines) was evaluated by estimating pollen germination rate in vitro. A minimum of ten flowers per genotype was collected from field-grown plants. Anthers were removed from flowers and placed in a desiccator. Pollen from fully dehisced anthers was distributed with a fine brush onto small Petri dishes (diameter: 5.5 cm) containing germination medium (Murashige and Skoog mineral medium with 3% sucrose and 0.8% agar, pH 5.7). These Petri dishes were placed inside larger Petri dishes (diameter: 9 cm) containing a moist piece of filter paper and incubated at 24°C in the dark for 24 h. Germination was quantified as the percentage of germinated pollen grains form a minimum of 600 grains counted.

The reproductive compatibility between the pollen donors and the recipient genotype in the T Plot were tested in vivo through directed hand crosses. The PC, P1, P7, CC, C1, LC and L8 lines were used as male parents in each single-pollination treatment. Hand pollinations were carried out in two years (2005 and 2006) by deposition of entire anthers on the stigmas of flowers from the clementine trees grown at the edge. The number of pollinated flowers per cross was 100. The fruits produced were collected at maturity and counted. Their seeds were extracted, counted and used in further analyses. For each pollination treatment, two measures of individual maternal fitness (“fruit set” and “seed set”) were used to determine the reproductive compatibility between the crossed lines. Fruit set was defined as the percentage of mature fruits produced from the total number of pollinated flowers. Seed set was defined as the number of viable seeds per fruit averaged over each treatment.

### Assessing the influence of other nearby pollen sources on PMTF frequencies

#### Potential pollen donor (PPD) genotypes in the neighboring plots

The role of the surrounding flora as an isolation barrier between transgenic pollen donors and recipients was examined through paternity analysis of the progeny from a subset of OP clementine trees for two years. For this purpose, surrounding citrus orchards were also taken into consideration as alternative pollen sources able to pollinate recipient plants in OP conditions. Thus, adult trees of any citrus genotype that was male fertile, cross compatible and synchronized in flowering with clementine at the study site (the T plot and neighboring plots within 100 m) were considered PPDs, as represented in Table 1. In the neighboring plots, named A and B, there were populations of adult citrus trees belonging to different breeding programs carried out at IVIA. Plot A consisted of a population of triploid hybrids as well as their diploid parental genotypes [29]. As triploid hybrids are sterile [30], only some of the diploid genotypes that are known to be cross-fertile with clementine mandarin were considered PPDs. Plot B was composed of a population of 477 hybrids belonging to a rootstock breeding program. These hybrids were randomly distributed within the plot, and all them were, in principle, potential pollinators of clementine.

**Table 1:** Potential pollen donor (PPD) genotypes present at the study site, including their abbreviation codes, population sizes (number of adult trees) and relative amounts.

Plot	PPD Genotype	code	Population size	Relative amount (%)
T	Pineapple sweet orange	P	24	3.80
	Carrizo citrange	C	24	3.80
	Mexican lime	L	24	3.80
A	Fortune mandarin	F	34	5.38
	Orlando tangelo	ORL	10	1.58
	Murcott mandarin	MU	7	1.11
	Nova tangor	N	6	0.95
	Ortanique tangor	ORT	6	0.95
	Willowleaf mandarin	MC	6	0.95
	Ellendale mandarin	E	6	0.95
	Kara mandarin	K	4	0.63
	Minneola tangelo	MI	4	0.63
B	King mandarin x *Poncirus trifoliata*	H1	202	31.96
	*C. volkameriana* x *Poncirus trifoliata*	H2	88	13.92
	Cleopatra mandarin x *Poncirus trifoliata*	H3	84	13.29
	Troyer citrange x Cleopatra mandarin	H4	77	12.18
	Troyer citrange x Willowleaf mandarin	H5	26	4.11

#### Molecular typing of progeny from OP recipients by microsatellite (SSR) analysis

Genomic DNA from progeny of a subset of OP recipient plants was subjected to SSR analysis to determine the pollen parentages of each hybrid seedling. Because there were no unique markers with total allelic differentiation among all PPD genotypes, we performed a multilocus paternity analysis. We chose 10 SSR markers that were highly polymorphic among PPD genotypes. These markers were *CI01G11, CIR07C07, CIR01E02* [31], *mest192* [32], *CIR01C06*, *CIR03C08* [33], *mest458*, *mest107*, *mest86* (Luro et al., unpublished) and *CAC23* [34]. PCRs with wellRED oligonucleotides (Sigma®), which use cyanine-based fluorescent dyes at the 5’end, were performed as described by [35] with slight modifications. An Eppendorf® Mastercycler ep gradient S was used with a reaction volume 15 µl, composed as follows: 0.8 U Taq polymerase (N.E.E.D.®), reaction buffer – 750 mM Tris HCl (pH 9), 50 mM KCl, 200 mM (NH_4_)_2_SO_4_, 0.001% BSA, 0.1 mM of each dNTP, 5 mM MgCl_2_, 3 µM of each primer, and 30 ng DNA. The following PCR program was used: 5 min at 94°C; 40 cycles of 30 sec at 94°C, 1 min at 50–55°C and 30 sec at 72°C; final elongation 10 min at 72°C. After performing the PCR, genetic analysis was performed in a capillary-array sequencer CEQTM 800 System (Beckman Coulter_ Inc., Fullerton, CA), and the results were analyzed with Genome- LabTM GeXP Genetic Analysis System software.

#### Paternity assignment

Paternity analysis was performed based on SSR genotyping, using a simple exclusion approach [36]. When the paternal allele(s) at a locus could be inferred from the observed progeny and maternal genotype, then all PPDs that lacked the allele(s) were excluded. This process was repeated for each locus, until all PPDs could be excluded except one. In some cases, it was not feasible to assign a single PPD even after the hybrid was analyzed for all the 10 markers. In these cases, phenotypical traits, such as leaf morphology (trifoliate vs. monofoliate) and GUS expression, were considered for discriminating among different ambiguously assigned PPDs.

#### Pollen competition studies

To clarify the mechanisms of isolation by which other PPDs at the study site limited PMTF frequencies, the pollen competition capacity of one of the PPDs displaying higher mating success in OP conditions (H3 in Table 1) was compared to that of one transgenic PPD of plot T (P1) by mixed pollination treatments over two years (2006–2007). P1 was chosen as the competitor from plot T because it had displayed high cross-compatibility with clementine in single pollination treatments and had three copies of the *uidA* transgene [26], meaning that inheritance of this trait would be considerably high (theoretically 87.5%, assuming independency between loci). Mixed pollinations were carried out by depositing one entire anther from each genotype onto the stigmas of clementine flowers. Previously, to avoid the possible influence of pollen density effects [37], the number of pollen grains per anther for each genotype had been determined to ensure the deposition of approximately the same number of pollen grains. Likewise, differences in pollen viability between both genotypes were estimated by determining the percentage of pollen germination in vitro, as described above.

One hundred flowers were pollinated per year. The fruits produced were collected at maturity and counted. Their seeds were extracted, counted and tested for GUS expression. The siring success of transgenic pollen (P1) in the mixed pollination treatment was inferred from the GUS-positive frequency achieved in the tested progeny. We compared this GUS expression rate to that obtained in the progeny of single-pollination control treatments performed with P1.

### Data analyses

For the molecular validation of the PMTF assessment method, the χ^2^-test was performed. The minimum sample sizes of progeny required for this purpose in both years were calculated according to [38].

In single pollination treatments, separate multifactor analyses of variance (ANOVA) were conducted to examine the effects of “Variety” and “Genetic Modification” of the pollinator and their interaction on the variables “Fruit set” and “Seed set”. LSD multiple range tests were performed for separation of means. Before performing the analyses, Box-Cox transformations [39] were applied on both variables to fit the data to a normal distribution.

Data obtained from paternity analysis were used (1) to estimate the maximum reproductive success of each plot, calculated as the total percentage of progeny assigned; (2) to provide a spatial overview of the pollen dispersal patterns from the different plots by performing radial graphs; (3) to examine the influence of the proximity of plot B in the mating chance of the rest of pollen sources by drawing pollen dispersal curves with the percentage of pollination events unambiguously assigned to each plot as the y-axis and the distance from plot B as the x-axis; (4) to assess the possible relationship between the relative abundance of each PPD in plot B and their maximum mating success achieved. Simple regression analyses were used to model the relationships between the variables for (3) and (4).

All statistical analyses were performed using STATGRAPHICS Plus 5.1.

## Results

### PMTF frequencies from three different citrus genotypes were unexpectedly very low in contiguous recipient trees

PMTF frequencies found at the study site showed that the percentage of transgenic seeds in self-incompatible clementine fruits was consistently very low (between 0.17% and 2.86%) (Table 2), taking into account the proximity of transgenic pollen donors to the recipient trees. As the numbers of flowers and bee pollinators were usually very high in the spring (Fig. S1), the average seed production in OP recipient trees was also high, as expected (Table 2). This high production allowed us to analyze many seeds (ranging from 603 to 2990) each year by histochemical GUS assays. This high number of seeds analyzed, together with the seven consecutive years of assessment, provided strong confidence to our results.

**Table 2:** The pollen-mediated transgene flow (PMTF) frequencies for seven years as determined by testing seeds from open-pollinated recipient trees for GUS expression.

Year	Number of seeds			PMTF (%)
	per fruit (seed set mean ± SE)	tested	GUS positive	
2001	7.91±0.63	2990	5	0.17
2002	1.21±0.12	1359	13	0.96
2003	2.68±0.25	2171	9	0.41
2004	0.80±0.12	603	5	0.67
2005	4.68±0.34	2619	75	2.86
2006	2.67±0.20	1573	22	1.39
2007	3.43±0.27	1398	29	2.18

Next, we decided to validate the method used and to investigate whether low/silenced GUS expression in seeds could be contributing to the low PMTF frequency observed. A total of 224 hybrid seedlings from 12 recipient trees in 2005 and 140 seedlings from 9 recipient trees in 2006 were tested for GUS expression in the leaves and *uidA* integration. Sample sizes used exceeded the minimum required to statistically represent the population at 95% confidence with an acquired precision error of ≤3%. The PMTF frequencies obtained from analyzing GUS expression in seedlings were 2.86% in 2005 and 1.39% in 2006 (Table 3). Moreover, PCR analyses confirmed, at the molecular level, the transgenic nature of all GUS-positive seedlings and dismissed the presence of transgene-silencing in GUS-negative seedlings without exception (Table 3). When comparing these results with those obtained previously for GUS expression in seeds in the same years (Table 1), a χ^2^-test showed no statistically significant differences between the frequencies for either of the two years at the 95% confidence level, indicating that the hybrid seed identification system used during the seven years of assessment to determine PMTF frequency was reliable.

**Table 3:** Molecular validation of the pollen-mediated transgene flow (PMTF) assessment method by testing seedlings from a subset of open-pollinated recipient trees during two years (2005 and 2006).

Year	clementine number	Number of seedlings			PMTF (%)^2^	χ^2^ value^3^
		Tested	Transgenic^1^	Trifoliated		
**2005**	2	21	0	13		
	8	21	1	0		
	14	3	0	1		
	20	30	1	6		
	25	6	0	1		
	27	15	4	9		
	29	11	0	0		
	35	5	0	2		
	42	15	1	4		
	48	36	0	9		
	53	27	0	4		
	55	34	0	6		
	**Total**	**224**	**7**	**55**	**3.12**	**0.024**
**2006**	2	6	0	0		
	6	11	0	2		
	20	2	0	0		
	27	15	2	1		
	30	18	0	1		
	36	18	0	0		
	42	30	0	2		
	50	34	0	1		
	55	6	0	5		
	**Total**	**140**	**2**	**12**	**1.42**	**0.001**

1 Number of transgenic seedlings was determined by GUS expression in leaves and confirmed by PCR analysis of the *uidA* transgene. False GUS negative seedlings were not found in any case.

2 PMTF frequency was calculated as the percentage of transgenic seedlings from the total number of seedlings analyzed per year.

3 For each year, χ^2^ tests were performed to compare the PMTF frequencies obtained by this method with the PMTF frequencies obtained by testing GUS expression in seeds (Table 2). The critical value for 1 *df* at a 95% confidence level is 3.84.

### Transgenic pollen donors and recipient trees showed flower synchrony and were cross compatible

**Figure 2:**
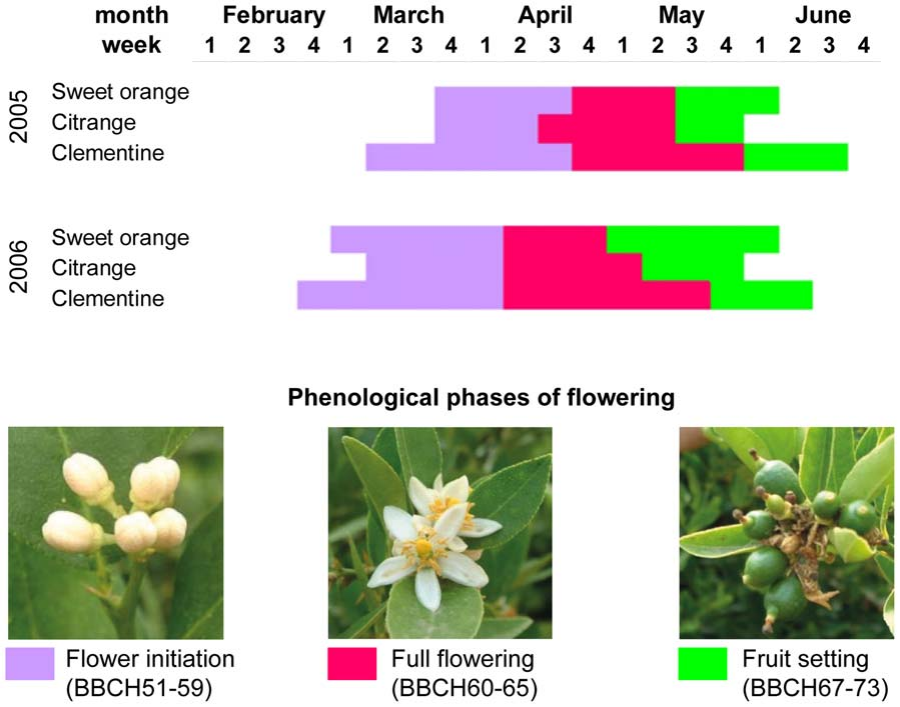
Phenological calendars of flowering for genotypes in plot T. Different phases of the bloom period for Pineapple sweet orange, Carrizo citrange and Clemenules clementine genotypes are represented by different colors. The overlap in the full-flowering phase (pink) determines the flowering synchrony between genotypes.

To discard the idea that low PMTF was due to asynchrony in flowering times between the transgenic pollen donors and the recipient clementine trees, phenological calendars of flowering were assessed and compared. The extent of the full flowering stage varied among citrus types and was longer in clementine trees. This stage lasted 3 and 4 weeks for Pineapple sweet orange and Carrizo citrange, respectively, while it lasted up to 6 weeks for Clemenules clementine. However, the full flowering phase of both pollen donor genotypes, though shorter, fully coincided with that of the recipient plants (Fig. 2).

The viability and capacity of fertilization of transgenic pollen was studied and compared with those of controls using in vitro and in vivo systems. In vitro studies of pollen viability showed that 1) germination rates varied among citrus types, reaching considerable high levels for sweet orange and citrange lines (about 50% and 70% on average, respectively) and 2) for each citrus type, pollen germination rates from transgenic lines did not differ from those of the correspondent controls (Fig S3). This demonstrates the absence of pleiotropic effects derived from the insertion of transgenes that affect negatively to pollen viability.

To check whether pollen donors from the T plot and recipient trees were cross compatible, hand pollinations were performed. As shown in Table S1, “Variety” was the most important factor determining cross-compatibility in directed crosses because it had effects on both variables (P-value = 0.0002 for fruit set; P-value = 0.0310 for seed set). Pineapple sweet orange and Carrizo citrange induced higher fruit set and seed set than Mexican lime (Fig. S4). The “GM” factor had no effect (*P*>0.05) on the variables investigated, indicating that transgenic trees were as compatible with recipients as the corresponding controls for the same background variety (Fig. S4).

### Influence of other nearby pollen sources on PMTF frequencies

#### Identification of specific, highly mating PPD types in the neighboring plots

**Figure 3:**
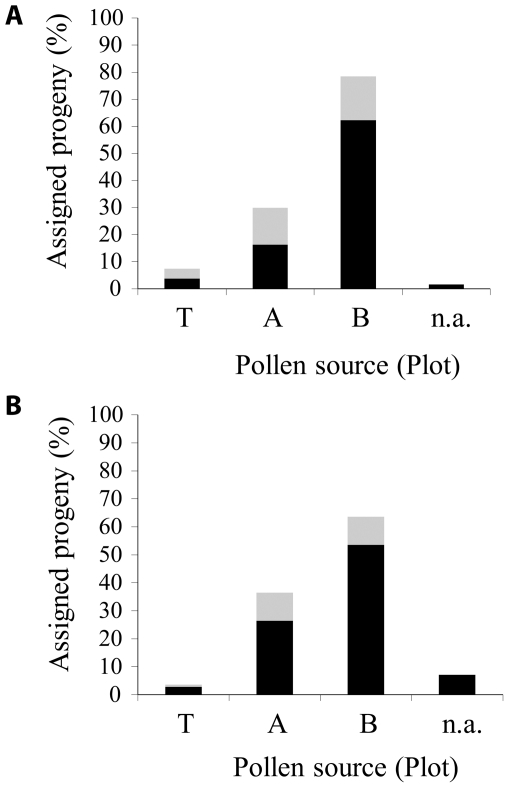
Maximum reproductive success assessed for each pollen source population in A) 2005 and B) 2006. Based on the classification of the pollination events made in Table 4, maximum reproductive success was estimated for each plot, as the percentage of pollination events unambiguously assigned (black color) plus the partial contributions of the percentages corresponding to pollination events ambiguously assigned (grey color). n.a., not assigned.

The analysis of GUS expression and leaf morphology in seedlings from a subset of OP recipient trees showed the presence of many trifoliate but GUS-negative hybrids (Table 3). Because transgenic Carrizo citrange trees were as cross compatible with clementines as their non-transgenic counterparts (Fig. S4), these results suggested that (trifoliate) neighbor trees from other surrounding plots were competing with trees from the T plot for pollination of recipient trees and likely interfering with the PMTF frequencies obtained. To identify the pollen source(s) that competed with pollen donors from the T plot under OP conditions, the DNA of 191 seedlings from 12 recipient trees and of 140 seedlings from 9 recipient trees was subjected to paternity analysis in 2005 and 2006, respectively. To this aim, marker profiles for each PPD genotype (or candidate father) from plots T and A were assessed as well as for the recipient (mother) genotype (Table S2). Because the PPD genotypes from plot B (reported in Table 1 as H1, H2, H3, H4 and H5) were F1 hybrids from a rootstock breeding program, their marker profiles in Table S2 corresponded to the alleles that could potentially be found in each F1 progeny, which were inferred from the known profiles of their parents. Then, hybrid seedlings were classified according to the source plot of their assigned parents (Table 4; Table S3 and Table S4). In this way, the percentage of progeny unambiguously assigned to a given plot was very high, 82.19% in 2005 and 79.28% in 2006, especially considering the close genetic background of many PPDs from the 3 plots. Moreover, the percentage of progeny that could not be assigned to any PPD (because their pollen parents within the population could not be assigned) was very low, 1.57% and 7.14% in 2005 and 2006, respectively. Based on these results, the analysis showed that the pollen source that had the highest reproductive success with recipient clementine trees was Plot B (78.5% in 2005 and 63.6% in 2006), followed by Plot A (29.8% in 2005 and 36.4% in 2006). Plot T showed the lowest reproductive success (7.4% in 2005 and 3.6% in 2006) (Fig. 3).

**Table 4:** Results of paternity assignment in progeny from open-pollinated recipients harvested in 2005 and 2006.

Number of pollen donor(s) assigned	Source of the pollen donor(s) assigned (Plot)	Category Name	Number of progeny placed within the class		Percent of progeny placed within the class	
			Year 2005	Year 2006	Year 2005	Year 2006
0	-	Not assigned	3	10	1.57	7.14
1	T	Unambiguously assigned T	7	3	3.66	2.14
1	A	Unambiguously assigned A	26	28	13.61	20.00
1	B	Unambiguously assigned B	45	47	23.56	33.57
>1	A	Unambiguously assigned A	5	7	2.62	5.00
>1	B	Unambiguously assigned B	74	26	38.74	18.57
>1	T/A	Ambiguously assigned T/A	0	1	0.00	0.71
>1	T/B	Ambiguously assigned T/B	5	1	2.62	0.71
>1	A/B	Ambiguously assigned A/B	24	12	12.57	8.57
>1	T/A/B	Ambiguously assigned T/A/B	2	2	1.04	1.43

All pollination events were categorized by the origin of the pollen donor(s) assigned according to microsatellite (SSR) genotyping, GUS expression and leaf morphology (trifoliate character).

#### Distance effect

**Figure 4:**
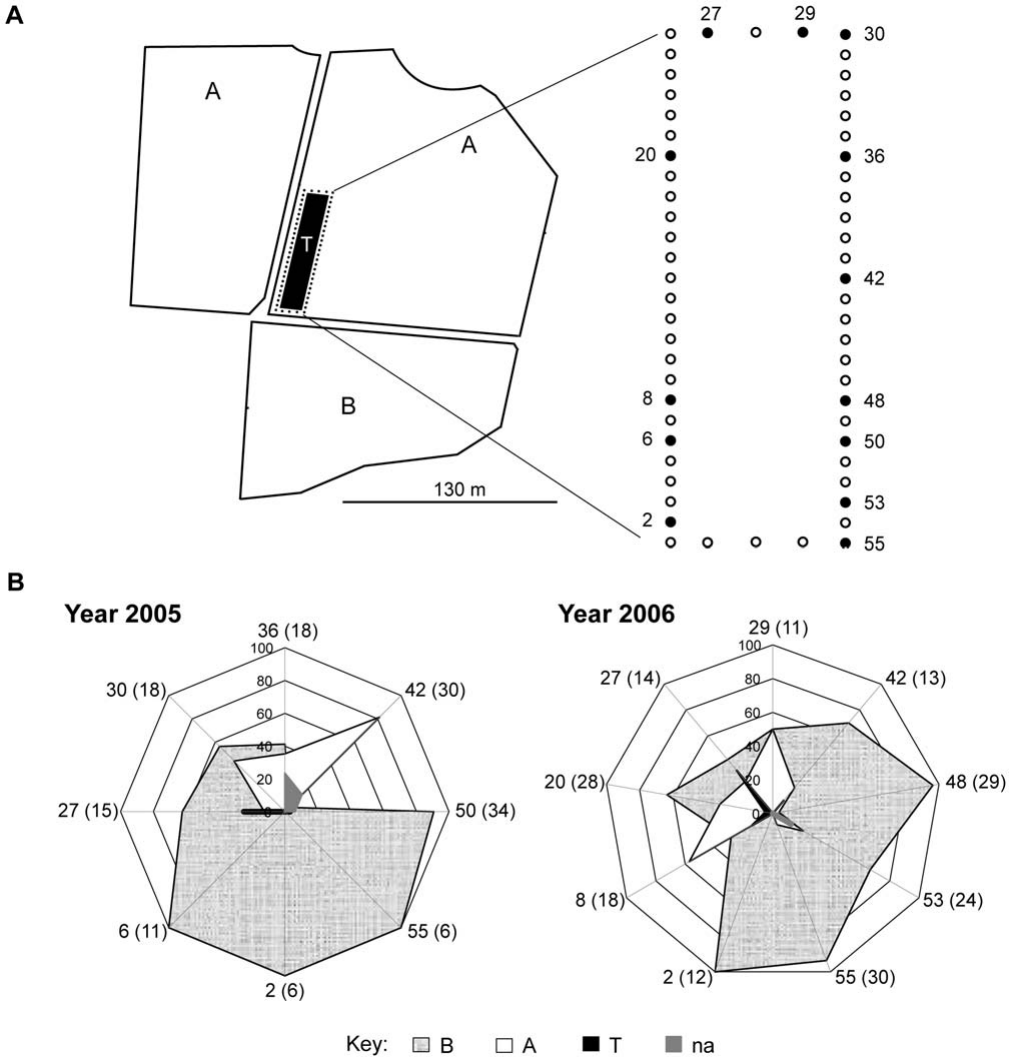
Schematic representation of pollen dispersal patterns at the study site. **A**) Map showing the relative location of recipients (dots) and pollen source populations (T, A and B plots). Recipient (mother) trees sampled in 2005 and/or 2006 whose progeny were analyzed for paternity assignment are represented by filled circles. **B**) Radial graphs represent the profiles of genotyped progeny from each mother tree. Numbers in the vertices indicate the recipient tree number followed by the total number of progeny seedlings analyzed from the mother tree (number in parentheses). The distribution of recipient trees in the vertices of the graph has been established according to their relative position in the field to accurately visualize the pollen dispersal patterns. The percentage of progeny from each corresponding recipient tree is represented on each radial axis by following categories: “B”, progeny unambiguously assigned to B; “A”, progeny unambiguously assigned to A; “T”, progeny unambiguously assigned to T; and “na”, progeny that could not be assigned to any PPD. Clementine plants producing an insufficient number of progeny seedlings were excluded from this study.

When considering the distance from recipient trees (Fig. 4), the frequency of mating events assigned to plot B was very high (almost 100%) in the progeny of recipient trees near that plot (see recipient numbers 2, 48, 53 and 55 for 2005 and recipient numbers 2, 6, 50 and 55 for 2006) and lower in recipients located at greater distances from the plot (see recipient numbers 20, 27 and 29 for 2005 and recipient numbers 27, 30 36, 42 for 2006), as expected. However, the extent of the mating capacity of plot B was considerably higher than that of competing plots because 50% of the mating events in the farthest recipient trees (see recipient numbers 20, 27 and 29 for 2005 and recipient numbers 27 and 30 for 2006) were clearly attributable to pollen from plot B (Fig. 4). Together, these results indicate that (1) the mating success of plot B was directly correlated with the distance to the recipient trees and (2) the mating capacity of plot B was able to explain (with 50% success) the parentage of hybrid seedlings from trees located at least 26 rows away.

The frequency of mating events assigned to plots T or A was null or very low in recipients near plot B and progressively increased with distance from that plot. Therefore, PPDs from plot B strongly limited the mating opportunities of the rest of PPDs from the study site, including those of the contiguous plot T. These results were reliable and indicate a consistent trend in pollen dispersal under our experimental conditions because the patterns were very similar in 2005 and 2006 (Fig. 4), likely also explaining the very low PMTF frequencies obtained during the seven years of the study (Table 2).

**Figure 5:**
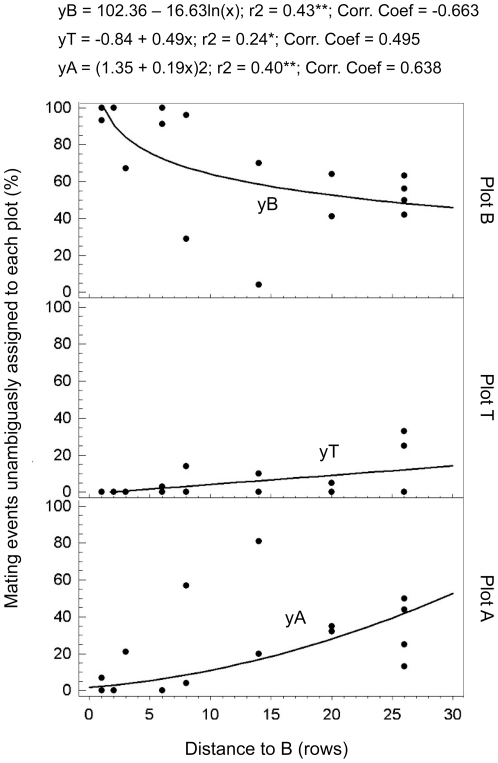
Pollen dispersal curves of each plot as a function of distance to plot B. Progeny from all recipient trees analyzed in 2005 and/or 2006 unambiguously assigned to each plot was divided into classes based on the distance between the (mother) recipient tree and plot B, measured in rows. Black dots represent the mating frequencies in each distance class as a proportion of all pollination events unambiguously assigned to this plot. Lines represent the curves fitted to regression models that best describe the relationship between mating frequencies and distance to Plot B for each pollen source population (*P<0.05; **P<0.01).

Pollen dispersal curves were performed to confirm the influence of the distance from plot B in the mating opportunity of each pollen source population. The logarithmic-X regression model showed that mating chance of plot B was strong and negatively correlated with the distance to B (R^2^ = 0.43; correlation coefficient  = -0.66). For plot T, the linear regression model showed a relatively weak positive relationship between the variables (R^2^ = 0.24; correlation coefficient  = 0.495). The square root regression model showed that the mating chance of plot A was moderately strong and positively correlated with the distance to B (R^2^ = 0.40; correlation coefficient  = 0.638) (Fig. 5).

#### Density effect

**Figure 6:**
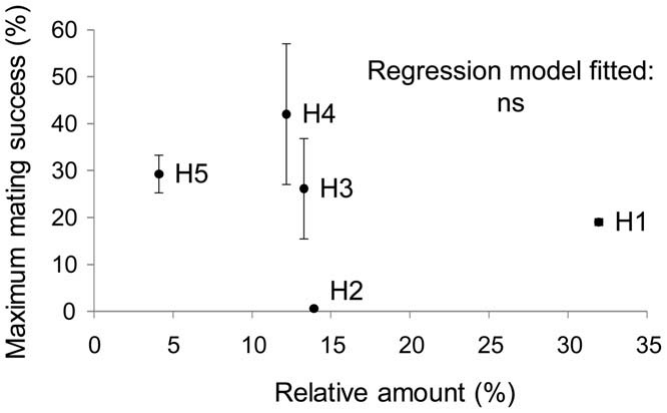
Density effect. Relationship between the maximum mating success achieved in 2005 and 2006 by each Potential Pollen Donor (PPD) of plot B (H1, H2, H3, H4 and H5) and its relative abundance at the study site (reported in Table 1). Black dots represent the proportion of mating events (unambiguously plus ambiguously) assigned to each PPD from plot B calculated over the total progeny unambiguously assigned to this plot and averaged between years. Bars represent standard errors. n.s., P>0.1 (not significant).

We attempted to determine whether the relative abundance of each PPD from plot B correlated with its mating success. As shown in Fig. 6, there was no statistically significant relationship (*P*>0.1) between these variables for any of the simple regression models fitted. Indeed, the most abundant PPDs, H1 and H2 (representing 31.96% and 13.92%, respectively, of the total number of PPDs at the study site) displayed low mating success compared to other less-abundant genotypes (such as H3, H4 and H5).

#### Pollen competition capacity/ability

The pollen competition capacity of H3 (a specific PPD from plot B that showed high mating success in OP conditions) was compared to that of P1 (a transgenic pollen donor of plot T) by mixed pollination treatments, with the aim of clarifying the mechanisms of isolation whereby the surrounding flora limited PMTF. Single pollinations of clementine flowers with P1 and PC, performed as controls, resulted in similar fruit set and seed set for both pollen donors (Table 5), indicating that the transgenic character of P1 did not affect its mating success. Moreover, 86% of the progeny seedlings from that cross were GUS positive, which fit well with expected transgene inheritance. In mixed pollinations with H3+P1, the percentage of GUS-positive progeny seedlings was extraordinarily reduced (5%) with respect to the expected rate if the pollen competition capacity of the two pollen donors were similar (43.75%). Additionally, mixed pollination resulted in a higher seed set (13.5±2.1) than single pollinations (6.8±1.7 for P1 and 7.2±1.9 for PC), indicating that (1) the H3 type strongly reduced the siring success of P1 and (2) it was much more efficient in cross pollination of recipient clementine trees than P1 or PC.

**Table 5:** Results of mixed-pollination treatments performed in 2006 and 2007 in comparison with single-pollination control treatments with PC and P1, including fruit set, seed set, and the percentage of GUS-positive (GUS+) seedlings in progeny as a parameter determining the siring success of P1.

Pollen source in pollination treatments		Fruit set (%)	Seed set (No. seeds/fruit)	GUS+ progeny (%)	Minimum no. of hybrid progeny analyzed per year
Mixed:	H3+P1	80.9±11.5	13.5±2.1	5.0±1.1	100
Single:	PC	78.5±17.7	7.2±1.9	0.0±0.0	340
	P1	68.0±12.7	6.8±1.7	86.0±3.4	222

## Discussion

We report here the first experimental field trial performed with transgenic citrus trees to study maximum transgene flow frequencies. With this aim, eight independent transgenic lines from three genetically diverse citrus types were used as transgenic pollen donors, and a non-transgenic self-incompatible citrus type planted along a contiguous edge was used as the recipient. The choice of a recipient unable to self-fertilize ensured a maximum outcrossing rate and facilitated the monitoring of transgene dispersal [40].

Pollination in most fruit trees, including citrus, is entomophilous [41], and honeybees are the predominant dispersal agents. Bees have the capacity to travel long distances (up to 3 km), but such long-distance flights are extremely rare in high-density plantings [42]. Consequently, as pollen-mediated gene flow in these species may be largely driven by the availability and foraging behavior of the pollinators [43], many studies have demonstrated that the maximum frequency of pollen-mediated gene flow between compatible and co-flowering crops occurred adjacent to the pollen source and typically decreased as the distance between crops increased, drastically decreasing 3 rows away (approximately 15 m) in the case of citrus [22].

In our experimental field, the spatial design, together with the lack of treatments against bees, allowed the maximum PMTF estimable in recipient trees under open-pollinated conditions to be achieved. However, contrary to our expectations, the data compiled during 7 years of assessment indicated that the rate of transgenic seeds from the edge trees was consistently very low. We decided to determine the factor/s that could have contributed to such results with the objective of proposing suitable containment measures applicable to future field trials with GM citrus and possibly other fruit tree crops.

The PMTF monitoring method used in this work was based on the expression of a tracer marker (*uidA*) in seeds. Visual markers have been extensively used in field trials because they make it relatively easy to follow the stability of transgene expression after outcrossing and accurately estimate gene flow [44]. To discard the possibility that transgene silencing and/or transgene loss in seeds from recipient trees could have masked the actual rate of transgene spread, we validated the monitoring method by analyzing transgene integration in hybrid seedlings during two consecutive years, and the results confirmed that only GUS-positive seeds carried the *uidA* transgene.

Next, we decided to examine isolation barriers that could have limited the mating opportunities between transgenic donors and recipients under our experimental conditions. Barriers to gene exchange between populations may arise through a variety of mechanisms. Pre-mating barriers, such as divergent flowering times and scarcity of flowers from the pollen source, could reduce opportunities for hybridization, thus limiting PMTF [45]. However, our phenological and visual surveys of flowering at the study site indicated that open flowers were highly abundant and synchronic in both transgenic pollen donor and recipient trees.

Reproductive barriers reduce gene flow between groups of organisms and act sequentially before and/or after mating [46]. It has been extensively reported that the potential gene flow from the transgenic pollen source to sympatric species is highly influenced by their reproductive compatibility, which can be measured by fruit set and seed set under controlled pollination conditions [47]. If the extent of reproductive compatibility between the transgenic source and overlapping genotypes were known in advance, it would represent an early ‘tier’ of risk assessment prior to the measurement of PMTF rates in experimental fields [48]. Single hand-pollination assays showed that the genetic background of the pollen source determined the extent of cross compatibility with the self-incompatible recipient. The importance of this factor has also been stressed in similar studies with other plant species, such as plum [49] and olive, [50] as well as in citrus [51]. As transgenic and control pollen donors produced viable pollen and were cross compatible with the recipient genotype in hand pollinations and the results were irrespective of the transgenic or non-transgenic nature of the pollen donor genotype, the very low rate of PMTF could not be attributed to low sexual compatibility between the source and sink nor to pleiotropic effects derived from expression of the transgenes.

Gene flow can also be influenced by the surrounding flora [52]. A diverse floral neighborhood may reduce conspecific pollen deposition by driving potential pollinators away or by increased heterospecific pollen deposition [53]. Therefore, a key factor that could greatly limit the gene flow between sexually compatible and flowering-synchronized species located at close proximity is the influence of the flowering environment, including conspecific and heterospecific co-flowering plants [54]. The presence of many seeds in fruits from self-incompatible OP recipient trees and the low PMTF obtained indicated effective pollen dispersal from other non-transgenic pollen source/s, most likely from citrus trees present in neighboring plots (A and B). Paternity analysis using molecular markers in the hybrid progeny from a subset of OP recipients confirmed the clear superiority in mating success for plot B. Moreover, the low mating success assigned to plot T (<8%) coincided with the very low PMTF rates observed along the seven consecutive years of assessment.

Additionally, pollen dispersal curves showed that the pollination competence of trees from plot B was so high that it strongly limited the mating opportunities of the other pollen sources within the study site, including those of the T plot, even when these were contiguous to the recipients. Furthermore, the mating competence of plot B decreased as the distance to the recipients increased, as expected based on the behavior of bees in citrus orchards [12].

Pollen dispersal curves of entomophilous plants are dependent on the foraging habits of the pollinators, which in turn are responsive to pollinator-linked pre-mating barriers, such as plant population size and density [43]. Bees are very sensitive to plant density and respond in a similar fashion regardless of the plant species involved. Density-dependent foraging distances and pollen dispersal may be a common feature for bees and bee-pollinated plants [41]. However, the relative abundances of each PPD from plot B did not correlate with their mating success efficiencies. Therefore, ecological or pollinator-linked pre-mating barriers were not sufficient to explain the results of the paternity analyses.

It has been suggested that reproductive barriers acting after pollination but before fertilization may play an important role in limiting gene flow [55]. If flowers receive more pollen grains from different pollen sources than the number of ovules they have, not every pollen grain will be able to sire a seed, and selection may occur during mating. This selection may involve discrimination between self and non-self pollen as well as discrimination among compatible donors, between too closely or too distantly related conspecifics, and among species [56]. Nonrandom mating among compatible mates at this level is of particular interest because it has the potential to produce sexual selection [57]–[59]. Such differential fertilization success often is stronger or exclusively observed when pollen from two species competes for fertilization [60]–[62]. Pollen competition is recognized as an important and common reproductive barrier [63], [64]. The mixed pollination treatments performed in this study demonstrated that a higher pollen competition capacity of H3 (a PPD from Plot B) compared to that of P1 (a pollen donor from Plot T) explained most of the mating superiority achieved by plot B in OP conditions (71.05% of hybrid progeny in OP conditions versus a maximum of 94.29% obtained in controlled hand pollinations), meaning that pollen competition may have greatly contributed to transgene confinement. Therefore, the presence of neighboring genotypes with very high pollen competition capacity is a crucial factor able to strongly limit PMTF between cross-compatible species when they have synchronized flowering and are planted at close proximity.

Based on these results, it is possible to propose transgene confinement measures that could be applicable to contiguous commercial plantings of citrus and may be extendible to other entomophilous fruit tree species, such as those from the genus *Malus*, *Pyrus*, *Cydonia*, *Eriobotrya* and *Prunus*:

1. Careful site examination and selection before the release of the GM crop. An essential first step is to determine the extent of reproductive compatibility and flowering synchrony between the transgene source and sympatric crops present at close proximity. If there were not previous information about these issues for the species/genotypes involved, it would be necessary to assess them before the release by performing controlled hand pollinations and phenological studies.
2. If the species involved were co-flowering and cross compatible, we propose the use of an external edge of trees from a non-GM pollen donor genotype showing pollen competition capacity clearly exceeding that of the transgenic pollen source. The use of a “strong pollinator” could serve as isolation barrier, acting as an alternative source for pollinators and/or as an effective competitor during the fertilization process with the transgenic pollen, and would make transgenic pollen escape practically nonexistent. The choice of the “strong pollinator” genotype would therefore depend on the species considered and could be based on the results obtained from mixed-pollination treatments carried out before the release.
3. We also propose the use of an external edge of trees from another non-GM genotype as an alternative pollen sink, as has previously been used by others [40]. The genotype chosen for this purpose should have several characteristics: flower synchrony with the transgenic genotype/s and the “strong pollinator”, production of high amounts of pollen to attract pollinators and male sterility or self-incompatibility. This edge of trees would facilitate estimating transgene flow frequencies over short distances.

## Supporting Information

Figure S1 **Representative pictures of plot T during the flowering period.** **A**) Picture showing the amount of flowers produced by transgenic pollen donor trees. **B**) Picture showing the presence of honeybees at the study site.
(DOC)

Figure S2 **Detection of transgenic hybrids in progeny from open-pollinated recipient trees.** **A**) Seed progeny screened for GUS expression. **B**) Seedling progeny cultivated on seedbeds in the greenhouse. **C**) Seedling progeny screened for GUS expression in the leaves. GUS+, GUS-positive. The scale bar on pictures **A**) and **C**) represents 10 mm.
(DOC)

Figure S3 **In vitro studies of pollen viability. A**) Effect of genotype on pollen germination rate. Bars represent means ± SE. **B**) Photographic views of pollen germination and tube growth (at 24°C after 24 h incubation in germination medium) from the P1 and H3 genotypes, chosen as competitors in mixed pollination treatments. Scale bars: 100 µm.
(DOC)

Figure S4 **In vivo studies of cross-compatibility.** The effect of different pollen donors on **A**) fruit set and **B**) seed set in directed crosses with recipient plants. The data are the means obtained in two years (2005 and 2006) ± standard error (SE) bars. The means with at least one common letter are not significantly different (*P*<0.05; LSD test).
(DOC)

Table S1 **ANOVA analysis for effects of Variety and Genetic Modification (GM) of the pollinator and their interaction on transformed versions of “Fruit set” and “Seed set” data obtained in single pollination treatments.**
(DOC)

Table S2 **Possible alleles of 10 SSR loci (markers) for each citrus genotype present at the study site and considered for paternity assignment, including clementine as known maternal genotype and all Potential Pollen Donors (PPD) as candidate fathers.**
(DOC)

Table S3 **Results of Paternity assignment in progeny from open-pollinated (OP) recipients harvested in 2005, according to microsatellite (SSR) genotyping, GUS expression and leaf morphology (trifoliate character).**
(DOC)

Table S4 **Results of paternity assignment in progeny from open-pollinated (OP) recipients harvested in 2006, according to microsatellite (SSR) genotyping, GUS expression and leaf morphology (trifoliate character).**
(DOC)
