# Supplementary material for: Pollen Competition as a Reproductive Isolation Barrier Represses Transgene Flow between Compatible and Co-Flowering Citrus Genotypes
Source: PLoS One. 2011 Oct 3;6(10):e25810. doi: 10.1371/journal.pone.0025810 (PMC3185051; doi:10.1371/journal.pone.0025810)

**Figure S1. Representative pictures of plot T during the flowering period.** **A**) Picture showing the amount of flowers produced by transgenic pollen donor trees. **B**) Picture showing the presence of honeybees at the study site.


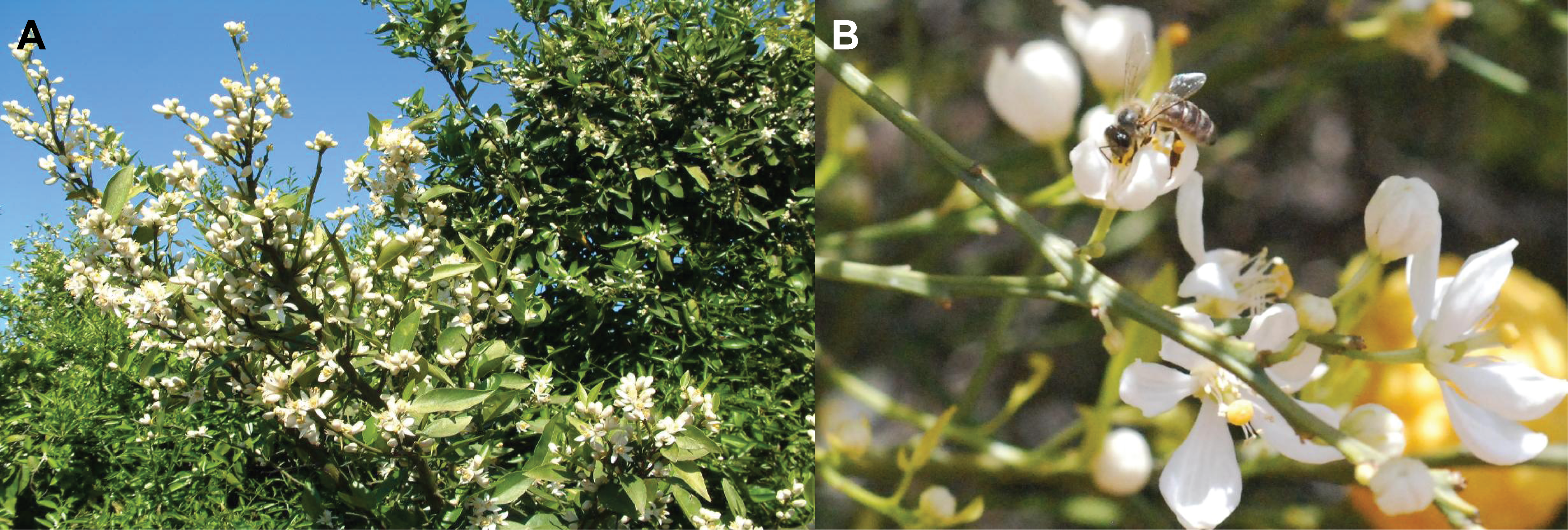

Supplement: Figure S1 — Representative pictures of plot T during the flowering period. A) Picture showing the amount of flowers produced by transgenic pollen donor trees. B) Picture showing the presence of honeybees at the study site. (DOC) [file pone.0025810.s001.doc]
