# Supplementary material for: Pollen Competition as a Reproductive Isolation Barrier Represses Transgene Flow between Compatible and Co-Flowering Citrus Genotypes
Source: PLoS One. 2011 Oct 3;6(10):e25810. doi: 10.1371/journal.pone.0025810 (PMC3185051; doi:10.1371/journal.pone.0025810)

**Figure S2. Detection of transgenic hybrids in progeny from open-pollinated recipient trees.** (**A**) Seed progeny screened for GUS expression. (**B**) Seedling progeny cultivated on seedbeds in the greenhouse. (**C**) Seedling progeny screened for GUS expression in the leaves. GUS+, GUS-positive. The scale bar on pictures **(A)** and **(C)** represents 10 mm.


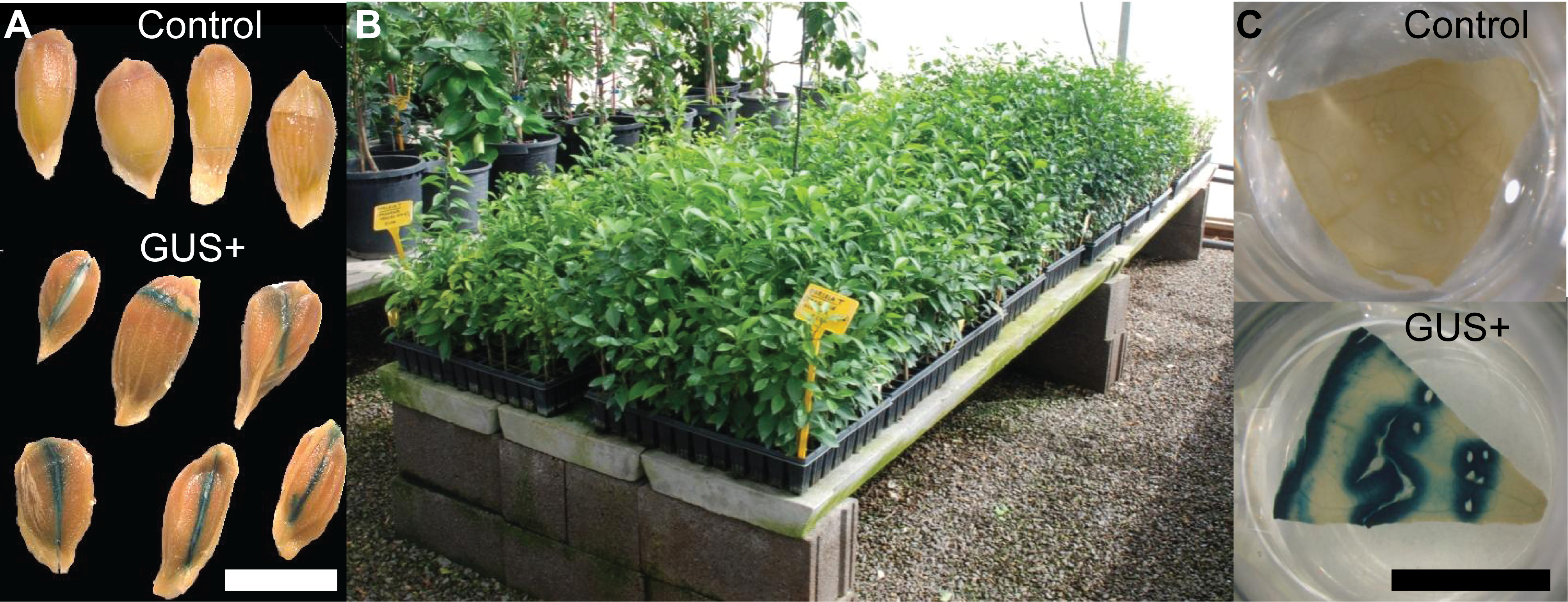

Supplement: Figure S2 — Detection of transgenic hybrids in progeny from open-pollinated recipient trees. A) Seed progeny screened for GUS expression. B) Seedling progeny cultivated on seedbeds in the greenhouse. C) Seedling progeny screened for GUS expression in the leaves. GUS+, GUS-positive. The scale bar on pictures A) and C) represents 10 mm. (DOC) [file pone.0025810.s002.doc]
