# Supplementary material for: Pollen Competition as a Reproductive Isolation Barrier Represses Transgene Flow between Compatible and Co-Flowering Citrus Genotypes
Source: PLoS One. 2011 Oct 3;6(10):e25810. doi: 10.1371/journal.pone.0025810 (PMC3185051; doi:10.1371/journal.pone.0025810)

**Figure S3. In vitro studies of pollen viability. (A**) Effect of genotype on pollen germination rate. Bars represent means ± SE*.* (**B**) Photographic views of pollen germination and tube growth (at 24°C after 24 h incubation in germination medium) from the P1 and H3 genotypes, chosen as competitors in mixed pollination treatments. Scale bars: 100µm.


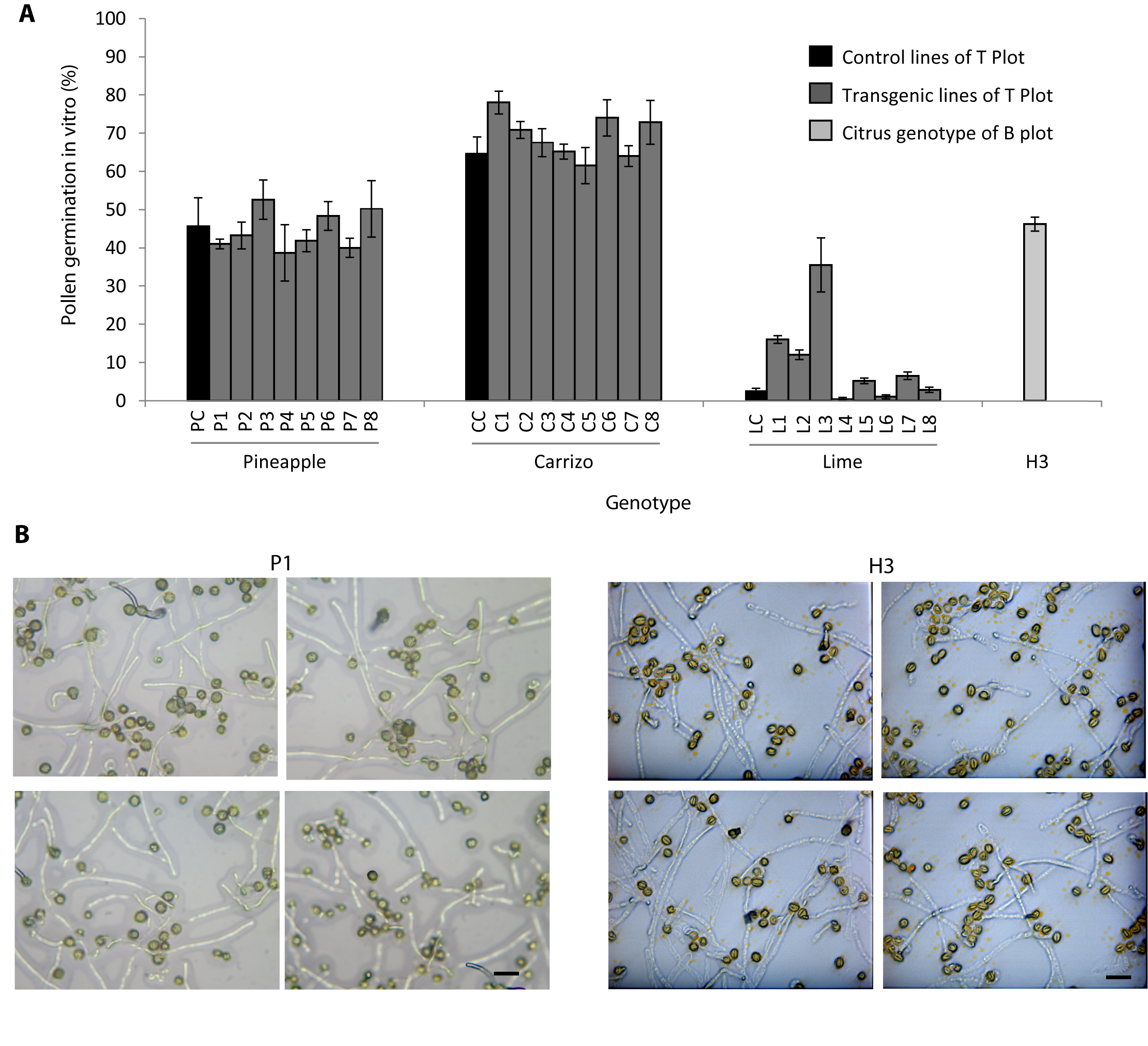

Supplement: Figure S3 — In vitro studies of pollen viability. A) Effect of genotype on pollen germination rate. Bars represent means ± SE. B) Photographic views of pollen germination and tube growth (at 24°C after 24 h incubation in germination medium) from the P1 and H3 genotypes, chosen as competitors in mixed pollination treatments. Scale bars: 100 µm. (DOC) [file pone.0025810.s003.doc]
