# Supplementary material for: Pollen Competition as a Reproductive Isolation Barrier Represses Transgene Flow between Compatible and Co-Flowering Citrus Genotypes
Source: PLoS One. 2011 Oct 3;6(10):e25810. doi: 10.1371/journal.pone.0025810 (PMC3185051; doi:10.1371/journal.pone.0025810)

**Figure S4. In vivo studies of cross-compatibility.** The effect of different pollen donors on **(A)** fruit set and **(B)** seed set in directed crosses with recipient plants. The data are the means obtained in two years (2005 and 2006) ± standard error (SE) bars. The means with at least one common letter are not significantly different (*P <* 0.05; LSD test).


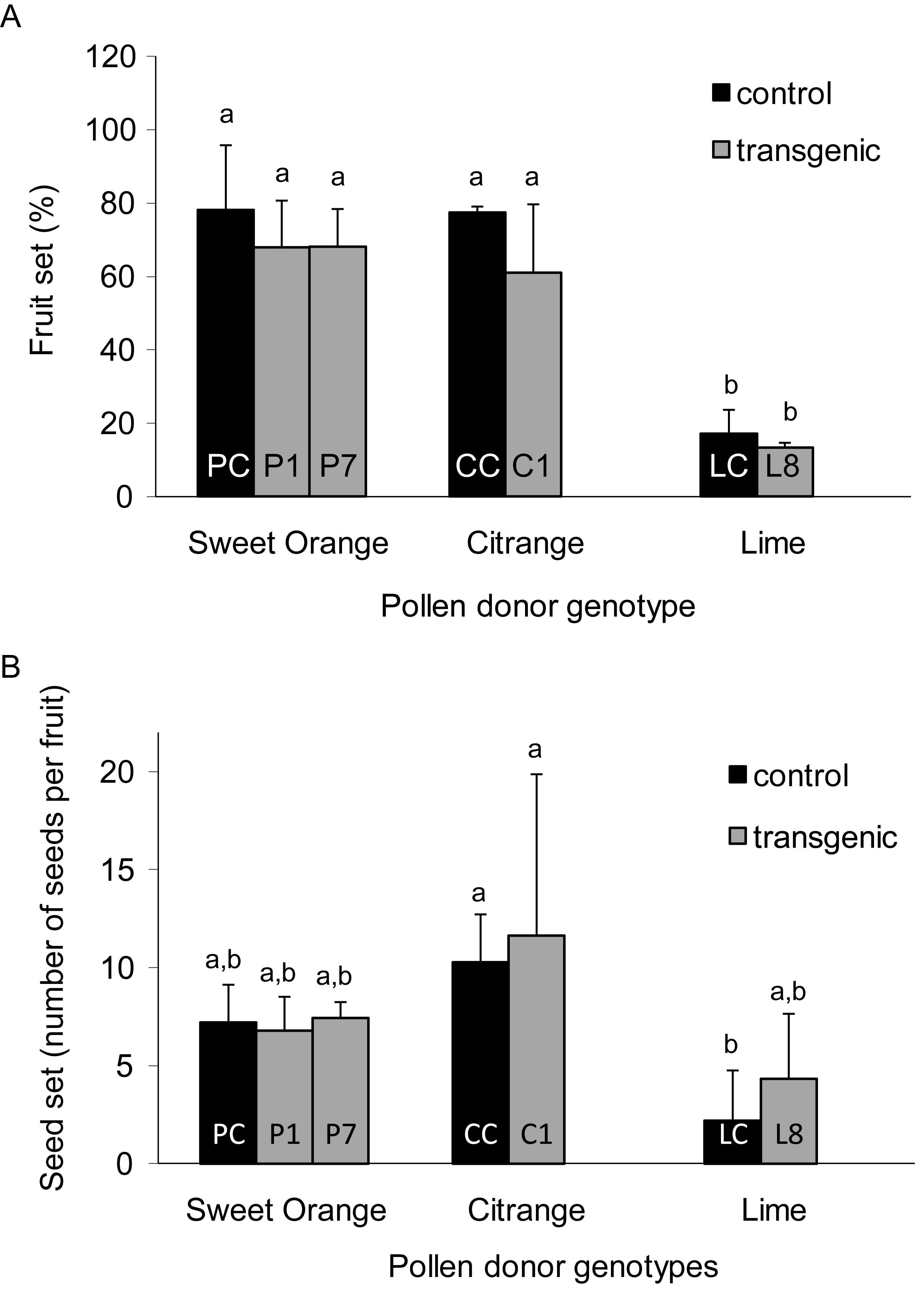

Supplement: Figure S4 — In vivo studies of cross-compatibility. The effect of different pollen donors on A) fruit set and B) seed set in directed crosses with recipient plants. The data are the means obtained in two years (2005 and 2006) ± standard error (SE) bars. The means with at least one common letter are not significantly different (P<0.05; LSD test). (DOC) [file pone.0025810.s004.doc]
