# Supplementary material for: Pollen Competition as a Reproductive Isolation Barrier Represses Transgene Flow between Compatible and Co-Flowering Citrus Genotypes
Source: PLoS One. 2011 Oct 3;6(10):e25810. doi: 10.1371/journal.pone.0025810 (PMC3185051; doi:10.1371/journal.pone.0025810)
Supplement: Table S1 — ANOVA analysis for effects of Variety and Genetic Modification (GM) of the pollinator and their interaction on transformed versions of “Fruit set” and “Seed set” data obtained in single pollination treatments. (DOC) [file pone.0025810.s005.doc]

**Table S1.** ANOVA analysis for effects of Variety and Genetic Modification (GM) of the pollinator and their interaction on transformed versions of “Fruit set” and “Seed set” data obtained in single pollination treatments.

| Variable | Source | df | MS | *F-*value | *P-*value |
| --- | --- | --- | --- | --- | --- |
| Fruit set | Variety | 2 | 4283.35 | 31.12 | 0.0002 |
|  | GM | 1 | 386.451 | 2.81 | 0.1323 |
|  | Variety x GM | 2 | 51.3173 | 0.37 | 0.7001 |
| Seed set | Variety | 2 | 56.0141 | 5.54 | 0.0310 |
|  | GM | 1 | 4.15599 | 0.41 | 0.5395 |
|  | Variety x GM | 2 | 2.41639 | 0.24 | 0.7930 |
