# Supplementary material for: Pollen Competition as a Reproductive Isolation Barrier Represses Transgene Flow between Compatible and Co-Flowering Citrus Genotypes
Source: PLoS One. 2011 Oct 3;6(10):e25810. doi: 10.1371/journal.pone.0025810 (PMC3185051; doi:10.1371/journal.pone.0025810)
Supplement: Table S2 — Possible alleles of 10 SSR loci (markers) for each citrus genotype present at the study site and considered for paternity assignment, including clementine as known maternal genotype and all Potential Pollen Donors (PPD) as candidate fathers. (DOC) [file pone.0025810.s006.doc]

**Table S2.** Possible alleles of 10 SSR loci (markers) for each citrus genotype present at the study site and considered for paternity assignment, including clementine as known maternal genotype and all Potential Pollen Donors (PPD) as candidate fathers.

| **Parental genotypes** | **PPD code** | ***CIR01C06*** | ***CIR07C07*** | ***CIR01E02*** | ***CIR01G11*** | ***mest458*** | ***CAC23*** | ***mest107*** | ***mest86*** | ***CIR03C08*** | ***mest192*** |
| --- | --- | --- | --- | --- | --- | --- | --- | --- | --- | --- | --- |
| Clemenules clementine (maternal genotype) | - | 134  166 | 227  239 | 155  167 | 103/109 | 214 | 248  251 | 176  184 | 112  128 | 207  225 | 227 |
| Pineapple sweet orange | P | 134  160 | 227 | 155  171 | 103/109 | 214  217 | 248  251 | 176  184 | 120  128 | 212  207 | 222  227 |
| Carrizo citrange | C | 146  160 | 212 | 170  171 | 103/109 | 213  217 | 245  248 | 173  176 | 118  120 | 212 | 227 |
| Mexican lime | L | 148  170 | 227 | 159 | 100/106  103/109 | 212  231 | 245  260 | 176  182 | 112 | 199  241 | 211  227 |
| Willowleaf mandarin | MC | 134  166 | 227  239 | 155  167 | 103/109 | 208  214 | 248  251 | 176 | 112  120 | 207  225 | 222  226 |
| Minneola tangelo | MI | 134  160 | 227 | 161  165 | 103/109 | 214  226 | 248  251 | 176  184 | 128 | 207 | 216  226 |
| Orlando tangelo | ORL | 132  134 | 225 | 161  165 | 103/109 | 214  226 | 248  251 | 176  184 | 128 | 207 | 202  222 |
| Ellendale mandarin | E | 132  134 | 227  239 | 155 | 103/109 | 214 | 248  251 | 176  184 | 112  128 | 207  225 | 216  226 |
| Murcot mandarin | MU | 132  160 | 225  237 | 155  161 | 100/106  103/109 | 214  226 | 248  251 | 176 | 112 | 207  228 | 216  230 |
| Ortanique tangor | ORT | 132  160 | 227  239 | 171 | 103/109 | 214  217 | 248  251 | 176  184 | 128 | 212  225 | 216  232 |
| Fortune mandarin | F | 134  166 | 239 | 165  167 | 103/109 | 214 | 248  251 | 176 | 112  128 | 225 | 216  226 |
| Kara mandarin | K | 132  146 | 233  237 | 155  157 | 100/106 | 217  226 | 248  251 | 176  184 | 112  120 | 222  225 | 222 |
| King mandarin x *Poncirus trifoliata* | H1 | 132  146  160 | 213  225  237 | 157  161  169  175 | 100/106  103/109 | 214  217  226 | 245  248  251 | 173  176  184 | 112  118  120 | 214  225  228 | 222  225  227  232 |
| *C. volkameriana* x *Poncirus trifoliata* | H2 | 146  170 | 212  235 | 160  176 | 103/109 | 206  213 | 245 | 173  176 | 112  118 | 212  225 | 230  242 |
| Cleopatra mandarin x *Poncirus trifoliata* | H3 | 132  146 | 212  237  241 | 161  170  176 | 100/106  103/109 | 208  213 | 245  248 | 173  176 | 112  118 | 214  222 | 222  225 |
| Troyer citrange x Cleopatra mandarin | H4 | 132  146  160  134 | 212  241  237  227 | 161  170  171  155  176 | 100/106  103/109 | 208  213  214  217 | 248  245  251 | 176  173  184 | 112  118  120 | 212  222 | 222  227 |
| Troyer citrange x Willowleaf mandarin | H5 | 146  166  134  160 | 227  212  239 | 155  170  171  176  167 | 103/109 | 208  217  213  214 | 248  251  245 | 173  176  184 | 112  118  120 | 212  207 | 222  226  227 |
