# Supplementary material for: Pollen Competition as a Reproductive Isolation Barrier Represses Transgene Flow between Compatible and Co-Flowering Citrus Genotypes
Source: PLoS One. 2011 Oct 3;6(10):e25810. doi: 10.1371/journal.pone.0025810 (PMC3185051; doi:10.1371/journal.pone.0025810)
Supplement: Table S3 — Results of Paternity assignment in progeny from open-pollinated (OP) recipients harvested in 2005, according to microsatellite (SSR) genotyping, GUS expression and leaf morphology (trifoliate character). (DOC) [file pone.0025810.s007.doc]

**Table S3.** Results of Paternity assignment in progeny from open-pollinated (OP) recipients harvested in 2005, according to microsatellite (SSR) genotyping, GUS expression and leaf morphology (trifoliate character).

| Number of pollen donor(s) assigned | Progeny of OP clementine (Seedling code) | Code(s) of pollen donor(s) assigned | Plot of pollen donor(s) assigned |
| --- | --- | --- | --- |
| 0 | 53.2 | - | Not assigned |
| 0 | 53.9 | - | Not assigned |
| 0 | 53.10 | - | Not assigned |
| 1 | 8.14 | P | T |
| 1 | 20.3 | P | T |
| 1 | 42.2 | C | T |
| 1 | 27.2 | C | T |
| 1 | 27.5 | P | T |
| 1 | 27.12 | P | T |
| 1 | 27.14 | C | T |
| 1 | 20.16 | H4 | B |
| 1 | 53.26 | H4 | B |
| 1 | 48.5 | H4 | B |
| 1 | 55.13 | H4 | B |
| 1 | 8.19 | H4 | B |
| 1 | 20.12 | H4 | B |
| 1 | 42.3 | H4 | B |
| 1 | 42.13 | H4 | B |
| 1 | 48.9 | H4 | B |
| 1 | 48.12 | H4 | B |
| 1 | 48.17 | H4 | B |
| 1 | 48.30 | H4 | B |
| 1 | 48.32 | H4 | B |
| 1 | 53.1 | H4 | B |
| 1 | 53.19 | H4 | B |
| 1 | 53.16 | H4 | B |
| 1 | 55.18 | H4 | B |
| 1 | 55.28 | H4 | B |
| 1 | 8.10 | H5 | B |
| 1 | 20.4 | H5 | B |
| 1 | 20.5 | H5 | B |
| 1 | 20.6 | H5 | B |
| 1 | 20.22 | H5 | B |
| 1 | 25.2 | H5 | B |
| 1 | 25.3 | H5 | B |
| 1 | 27.3 | H5 | B |
| 1 | 27.4 | H5 | B |
| 1 | 27.13 | H5 | B |
| 1 | 27.15 | H5 | B |
| 1 | 35.5 | H5 | B |
| 1 | 42.6 | H5 | B |
| 1 | 42.10 | H5 | B |
| 1 | 42.14 | H5 | B |
| 1 | 42.15 | H5 | B |
| 1 | 48.8 | H5 | B |
| 1 | 53.22 | H5 | B |
| 1 | 55.30 | H5 | B |
| 1 | 55.34 | H5 | B |
| 1 | 2.1 | H5 | B |
| 1 | 2.4 | H5 | B |
| 1 | 2.6 | H5 | B |
| 1 | 2.8 | H5 | B |
| 1 | 2.9 | H5 | B |
| 1 | 20.2 | H1 | B |
| 1 | 27.1 | H1 | B |
| 1 | 8.2 | MI | A |
| 1 | 8.11 | F | A |
| 1 | 8.20 | F | A |
| 1 | 8.21 | F | A |
| 1 | 14.1 | F | A |
| 1 | 20.8 | F | A |
| 1 | 53.17 | N | A |
| 1 | 20.14 | N | A |
| 1 | 53.4 | N | A |
| 1 | 20.24 | F | A |
| 1 | 20.25 | F | A |
| 1 | 29.1 | F | A |
| 1 | 29.4 | F | A |
| 1 | 29.5 | F | A |
| 1 | 29.11 | F | A |
| 1 | 35.1 | F | A |
| 1 | 35.3 | F | A |
| 1 | 42.9 | F | A |
| 1 | 42.12 | F | A |
| 1 | 55.29 | F | A |
| 1 | 53.5 | F | A |
| 1 | 20.27 | ORL | A |
| 1 | 20.10 | ORL | A |
| 1 | 27.9 | MU | A |
| 1 | 27.10 | MU | A |
| 1 | 27.11 | MU | A |
| >1 | 55.3 | H3, H4 | B |
| >1 | 55.5 | H3, H4 | B |
| >1 | 14.2 | H3, H4 | B |
| >1 | 14.3 | H3, H4 | B |
| >1 | 20.9 | H3, H4 | B |
| >1 | 20.18 | H3, H4 | B |
| >1 | 20.26 | H3, H4 | B |
| >1 | 29.3 | H3, H4 | B |
| >1 | 48.7 | H3, H4 | B |
| >1 | 48.11 | H3, H4 | B |
| >1 | 48.18 | H3, H4 | B |
| >1 | 48.19 | H3, H4 | B |
| >1 | 48.23 | H3, H4 | B |
| >1 | 48.25 | H3, H4 | B |
| >1 | 48.29 | H3, H4 | B |
| >1 | 48.36 | H3, H4 | B |
| >1 | 53.3 | H3, H4 | B |
| >1 | 53.6 | H3, H4 | B |
| >1 | 53.15 | H3, H4 | B |
| >1 | 53.20 | H3, H4 | B |
| >1 | 53.25 | H3, H4 | B |
| >1 | 55.7 | H3, H4 | B |
| >1 | 55.9 | H3, H4 | B |
| >1 | 55.10 | H3, H4 | B |
| >1 | 55.14 | H3, H4 | B |
| >1 | 55.16 | H3, H4 | B |
| >1 | 55.17 | H3, H4 | B |
| >1 | 55.20 | H3, H4 | B |
| >1 | 55.24 | H3, H4 | B |
| >1 | 55.26 | H3, H4 | B |
| >1 | 55.27 | H3, H4 | B |
| >1 | 55.31 | H3, H4 | B |
| >1 | 55.32 | H3, H4 | B |
| >1 | 55.33 | H3, H4 | B |
| >1 | 2.2 | H3, H4 | B |
| >1 | 53.8 | H3, H4, H1 | B |
| >1 | 55.21 | H3, H4, H1 | B |
| >1 | 20.15 | H3, H4, H1 | B |
| >1 | 29.2 | H3, H4, H1 | B |
| >1 | 29.10 | H3, H4, H1 | B |
| >1 | 48.10 | H3, H4, H1 | B |
| >1 | 48.13 | H3, H4, H1 | B |
| >1 | 48.14 | H3, H4, H1 | B |
| >1 | 48.16 | H3, H4, H1 | B |
| >1 | 48.21 | H3, H4, H1 | B |
| >1 | 48.26 | H3, H4, H1 | B |
| >1 | 48.31 | H3, H4, H1 | B |
| >1 | 48.33 | H3, H4, H1 | B |
| >1 | 48.34 | H3, H4, H1 | B |
| >1 | 53.13 | H3, H4, H1 | B |
| >1 | 53.18 | H3, H4, H1 | B |
| >1 | 53.21 | H3, H4, H1 | B |
| >1 | 53.23 | H3, H4, H1 | B |
| >1 | 55.11 | H3, H4, H1 | B |
| >1 | 55.15 | H3, H4, H1 | B |
| >1 | 55.25 | H3, H4, H1 | B |
| >1 | 2.5 | H3, H4, H1 | B |
| >1 | 2.7 | H3, H4, H1 | B |
| >1 | 2.10 | H3, H4, H1 | B |
| >1 | 2.12 | H3, H4, H5, H1 | B |
| >1 | 48.2 | H3, H4, H5 | B |
| >1 | 48.22 | H3, H4, H5 | B |
| >1 | 55.12 | H3, H4, H5 | B |
| >1 | 55.4 | H3, H2, H1 | B |
| >1 | 29.9 | H4, H1 | B |
| >1 | 20.28 | H4, H5 | B |
| >1 | 2.11 | H4, H5 | B |
| >1 | 2.3 | H4, H5 | B |
| >1 | 20.11 | H4, H5 | B |
| >1 | 20.21 | H4, H5 | B |
| >1 | 35.2 | H4, H5 | B |
| >1 | 27.7 | H4, H5 | B |
| >1 | 42.1 | H4, H5 | B |
| >1 | 53.7 | H4, H5 | B |
| >1 | 48.3 | E, ORT, N | A |
| >1 | 20.1 | E, ORT, N | A |
| >1 | 53.12 | E, ORT, N | A |
| >1 | 53.27 | E, ORT, N | A |
| >1 | 55.2 | E, ORT, N | A |
| >1 | 42.8 | C, H4 ,H5, H1 | T / B |
| >1 | 27.8 | C, H4, H5 | T / B |
| >1 | 48.4 | C, H4, H5 | T / B |
| >1 | 55.8 | C, H3, H4, H5, H1 | T / B |
| >1 | 8.6 | P, H5 | T / B |
| >1 | 20.7 | H1, ORL,MU | A / B |
| >1 | 20.19 | H1, ORL,MU | A / B |
| >1 | 20.20 | H1, ORL,MU | A / B |
| >1 | 20.23 | H1, ORL,MU | A / B |
| >1 | 20.29 | H1, ORL,MU | A / B |
| >1 | 20.30 | H1, ORL,MU | A / B |
| >1 | 48.20 | H1, MU | A / B |
| >1 | 25.1 | H5, MC, F, N | A / B |
| >1 | 25.5 | H5, MC, F, N | A / B |
| >1 | 29.6 | H5, MC, F, N | A / B |
| >1 | 29.8 | H5, MC, F, N | A / B |
| >1 | 42.7 | H5, MC, F, N | A / B |
| >1 | 55.6 | H5, MC, F, N | A / B |
| >1 | 8.1 | H5, MC F, N | A / B |
| >1 | 8.8 | H5, MC, F, N | A / B |
| >1 | 8.13 | H5, MC, E, F | A / B |
| >1 | 25.6 | H5, MC | A / B |
| >1 | 8.12 | H5, MC | A / B |
| >1 | 42.11 | H5, MC | A / B |
| >1 | 8.4 | H5, MC | A / B |
| >1 | 8.3 | H4, H5, MC | A / B |
| >1 | 8.9 | H4, H5, MC | A / B |
| >1 | 8.16 | H4, H5, MC | A / B |
| >1 | 29.7 | H4, H5, MC | A / B |
| >1 | 8.15 | P, H4,H5, ORT | T / A / B |
| >1 | 8.5 | P, H4,H5, ORT | T / A / B |
