# Supplementary material for: Pollen Competition as a Reproductive Isolation Barrier Represses Transgene Flow between Compatible and Co-Flowering Citrus Genotypes
Source: PLoS One. 2011 Oct 3;6(10):e25810. doi: 10.1371/journal.pone.0025810 (PMC3185051; doi:10.1371/journal.pone.0025810)
Supplement: Table S4 — Results of paternity assignment in progeny from open-pollinated (OP) recipients harvested in 2006, according to microsatellite (SSR) genotyping, GUS expression and leaf morphology (trifoliate character). (DOC) [file pone.0025810.s008.doc]

**Table S4.** Results of paternity assignment in progeny from open-pollinated (OP) recipients harvested in 2006, according to microsatellite (SSR) genotyping, GUS expression and leaf morphology (trifoliate character).

| Number of pollen donor(s) assigned | Progeny of OP clementine (Seedling code) | Code(s) of pollen donor(s) assigned | Plot of pollen donor(s) assigned |
| --- | --- | --- | --- |
| 0 | 20.6 | - | Not assigned |
| 0 | 36.14 | - | Not assigned |
| 0 | 36.2 | - | Not assigned |
| 0 | 36.4 | - | Not assigned |
| 0 | 36.5 | - | Not assigned |
| 0 | 42.15 | - | Not assigned |
| 0 | 42.27 | - | Not assigned |
| 0 | 42.35 | - | Not assigned |
| 0 | 50.29 | - | Not assigned |
| 0 | 50.6 | - | Not assigned |
| 1 | 27.4 | P | T |
| 1 | 27.5 | P | T |
| 1 | 50.24 | L | T |
| 1 | 30.11 | H1 | B |
| 1 | 36.8 | H1 | B |
| 1 | 50.18 | H1 | B |
| 1 | 50.37 | H1 | B |
| 1 | 55.8 | H1 | B |
| 1 | 6.7 | H1 | B |
| 1 | 30.13 | H2 | B |
| 1 | 2.2 | H3 | B |
| 1 | 2.4 | H3 | B |
| 1 | 2.3 | H4 | B |
| 1 | 2.6 | H4 | B |
| 1 | 20.1 | H4 | B |
| 1 | 30.5 | H4 | B |
| 1 | 36.9 | H4 | B |
| 1 | 50.21 | H4 | B |
| 1 | 50.28 | H4 | B |
| 1 | 50.36 | H4 | B |
| 1 | 50.38 | H4 | B |
| 1 | 50.7 | H4 | B |
| 1 | 50.8 | H4 | B |
| 1 | 50.9 | H4 | B |
| 1 | 6.11 | H4 | B |
| 1 | 6.12 | H4 | B |
| 1 | 6.16 | H4 | B |
| 1 | 2.9 | H5 | B |
| 1 | 27.11 | H5 | B |
| 1 | 27.2 | H5 | B |
| 1 | 27.8 | H5 | B |
| 1 | 27.9 | H5 | B |
| 1 | 30.1 | H5 | B |
| 1 | 30.12 | H5 | B |
| 1 | 30.15 | H5 | B |
| 1 | 30.18 | H5 | B |
| 1 | 36.1 | H5 | B |
| 1 | 36.10 | H5 | B |
| 1 | 36.18 | H5 | B |
| 1 | 42.19 | H5 | B |
| 1 | 50.15 | H5 | B |
| 1 | 50.2 | H5 | B |
| 1 | 50.23 | H5 | B |
| 1 | 50.3 | H5 | B |
| 1 | 50.30 | H5 | B |
| 1 | 50.31 | H5 | B |
| 1 | 55.10 | H5 | B |
| 1 | 55.14 | H5 | B |
| 1 | 55.4 | H5 | B |
| 1 | 6.6 | H5 | B |
| 1 | 6.8 | H5 | B |
| 1 | 30.6 | F | A |
| 1 | 36.13 | F | A |
| 1 | 36.19 | F | A |
| 1 | 42.11 | F | A |
| 1 | 42.17 | F | A |
| 1 | 42.2 | F | A |
| 1 | 42.3 | F | A |
| 1 | 30.17 | MI | A |
| 1 | 30.7 | MI | A |
| 1 | 30.9 | MI | A |
| 1 | 36.17 | MI | A |
| 1 | 42.10 | MI | A |
| 1 | 42.14 | MI | A |
| 1 | 42.20 | MI | A |
| 1 | 42.28 | MI | A |
| 1 | 42.31 | MI | A |
| 1 | 42.7 | MI | A |
| 1 | 42.33 | MI | A |
| 1 | 30.21 | N | A |
| 1 | 36.11 | N | A |
| 1 | 36.20 | N | A |
| 1 | 42.18 | N | A |
| 1 | 42.21 | N | A |
| 1 | 42.24 | N | A |
| 1 | 42.32 | N | A |
| 1 | 42.8 | N | A |
| 1 | 36.12 | N | A |
| 1 | 42.26 | N | A |
| 1 | 27.6 | ORL | A |
| >1 | 2.1 | H1, H4 | B |
| >1 | 50.1 | H1, H4 | B |
| >1 | 50.19 | H1, H4 | B |
| >1 | 50.26 | H1, H4 | B |
| >1 | 50.27 | H1, H4 | B |
| >1 | 6.13 | H1, H4 | B |
| >1 | 6.3 | H1, H4 | B |
| >1 | 30.23 | H1, H4 | B |
| >1 | 36.3 | H1, H4 | B |
| >1 | 50.4 | H1, H4 | B |
| >1 | 27.7 | H1, H3, H4 | B |
| >1 | 30.22 | H1, H3, H4 | B |
| >1 | 36.16 | H1, H3, H4 | B |
| >1 | 50.10 | H1, H3, H4 | B |
| >1 | 50.11 | H1, H3, H4 | B |
| >1 | 50.12 | H1, H3, H4 | B |
| >1 | 50.16 | H1, H3, H4 | B |
| >1 | 50.20 | H1, H3, H4 | B |
| >1 | 50.22 | H1, H3, H4 | B |
| >1 | 50.25 | H1, H3, H4 | B |
| >1 | 50.34 | H1, H3, H4 | B |
| >1 | 50.35 | H1, H3, H4 | B |
| >1 | 55.12 | H1, H3, H4 | B |
| >1 | 55.15 | H1, H3, H4 | B |
| >1 | 6.10 | H1, H3, H4 | B |
| >1 | 6.14 | H1, H3, H4 | B |
| >1 | 6.15 | H1, H3, H4 | B |
| >1 | 30.24 | MI, F | A |
| >1 | 42.16 | MI, F | A |
| >1 | 42.30 | MI, F | A |
| >1 | 42.4 | MI, F | A |
| >1 | 42.6 | MI, F | A |
| >1 | 42.34 | MI, ORL | A |
| >1 | 30.3 | ORL, MU | A |
| >1 | 50.14 | C, H4, H5 | T / B |
| >1 | 50.17 | P, MI, E, ORT | T / A |
| >1 | 30.2 | H1, ORL, MU | A / B |
| >1 | 42.37 | H4, N | A / B |
| >1 | 27.14 | H5, MC | A / B |
| >1 | 27.15 | H5, MC | A / B |
| >1 | 27.16 | H5, MC | A / B |
| >1 | 27.3 | H5, MC | A / B |
| >1 | 42.36 | H5, MC, F | A / B |
| >1 | 27.13 | H5, MC, F, N | A / B |
| >1 | 27.20 | H5, MC, F, N | A / B |
| >1 | 30.14 | H5, MC, F, N | A / B |
| >1 | 36.15 | H5, MC, F, N | A / B |
| >1 | 42.5 | H5, MC, N | A / B |
| >1 | 27.10 | H5, P, MC | T / A / B |
| >1 | 42.9 | H5, P, MC, E, F | T / A / B |
